# Mechanical Properties and Microstructure of Class C Fly Ash-Based Geopolymer Paste and Mortar

**DOI:** 10.3390/ma6041485

**Published:** 2013-04-09

**Authors:** Xueying Li, Xinwei Ma, Shoujie Zhang, Enzu Zheng

**Affiliations:** 1 School of Civil Engineering, Harbin Institute of Technology, Harbin 150006, China; E-Mail: hitzez@163.com; 2 Department of Civil Engineering, Harbin Institute of Technology at WeiHai, Weihai 264209, China; 3 Heilongjiang Provincial Hydraulic Research Institute, Harbin 150080, China; E-Mail: zsj888265@sohu.com

**Keywords:** geopolymer, class C fly ash, compressive strength, workability, microstructure

## Abstract

This paper presents workability, compressive strength and microstructure for geopolymer pastes and mortars made of class C fly ash at mass ratios of water-to-fly ash from 0.30 to 0.35. Fluidity was in the range of 145–173 mm for pastes and 131–136 mm for mortars. The highest strengths of paste and mortar were 58 MPa and 85 MPa when they were cured at 70 °C for 24 h. In XRD patterns, unreacted quartz and some reacted product were observed. SEM examination indicated that reacted product has formed and covered the unreacted particles in the paste and mortar that were consistent with their high strength.

## 1. Introduction

Recently, a new alternative binder termed geopolymer has emerged in the field of construction and building materials. Geopolymer is well known for its excellent properties such as good fire and acid resistance, high compressive strength, low shrinkage, and solidification of heavy metal wastes, *etc.* [1,2]. This material is usually based on an alumino–silicate precursor activated in a concentrated alkali hydroxide solution, to which is often added alkali silicate to control the final chemical composition. At present, efforts have been made to investigate geopolymer prepared using fly ash. However, work on fly ash geopolymer so far has been based on class F fly ash [3,4,5], and few published papers have described geopolymer made using class C fly ash. The main distinction between class F fly ash and class C fly ash is that the latter contains a higher amount of calcium. Previous researchers pointed out that literature was generally focused on the activation of low calcium fly ashes, in which the calcium was more seen as a contaminant producing different hydrate [6,7] assemblages that may cause decrease in strength [8] and reduced rate of reaction [9]. A few studies, on the other hand, have concluded that class C fly ash has both pozzolantic and cementitious properties [10,11]. And calcium content has significant influence on the properties of the fresh mixture as well as the properties of the final hardened product [12,13] and may lead to the formation of calcium silicate hydrate compounds in addition to the geopolymer gel products, augmenting the mechanical strength of the hardened matrix [14]. In geopolymer made using class C fly ash, curing at ambient and elevated temperature could produce higher strength compared to geopolymer made using class F fly ash [15].

Geopolymer have been manufactured as paste, mortar and concrete. In previous studies, compressive strength and Young’s modulus of fly ash-based geopolymer did not change significantly between paste and mortar [5,16]. However, in mortar, compressive strength depends on the strength of the geopolymeric gel, the interfacial bonding between the geopolymeric gel and aggregate and the aggregate itself [5]. Moreover, any partial reaction of the surfaces of siliceous aggregates with the alkali silicate solution may form additional reaction products surrounding the aggregate particles that may contribute to its strength [17,18]. Thus, the properties of geopolymer paste and mortar differ to some extent.

This study considers mechanical properties and microstructure of class C fly ash-based geopolymer paste and mortar.

## 2. Experimental Test

### 2.1. Materials

Class C fly ash (CFA) used in this study was obtained from Harbin Acheng Suibao Thermoelectric Power Plant, China. Chemical composition of fly ash precursor is 48.2% SiO_2_, 18.4% Al_2_O_3_ 19.6% CaO, 3.7% Fe_2_O_3_, 1.1 MgO, 1.7% SO_3_, 5.2% free-CaO.

**Figure 1:**
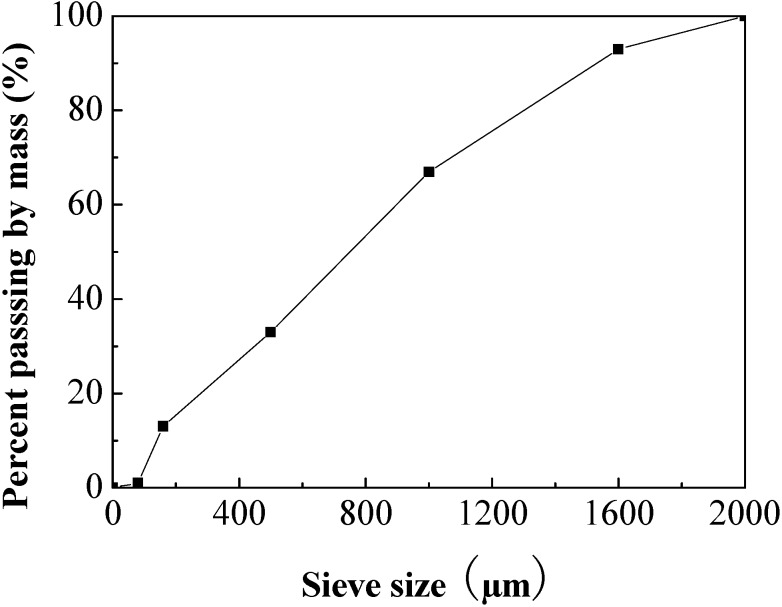
Grading curve of sand aggregate.

The fly ash was mechanically activated in a steel ball mill for 1 hour. Its specific surface area was 490 m^2^/kg. Alkaline activators in the investigation were sodium silicate which contained Na_2_O and SiO_2_ and solid content of 41% from Julide Chemical Co., Langfang, China, and solid content of 41% and sodium hydroxide solution prepared from analytical grade sodium hydroxide pellets. Mineralogical composition of the sand aggregate used in this study is silica produced in Xiamen, China. The granulometry distribution of the sand obtained by sieve analysis is shown in Figure 1.

### 2.2. Mixture Design and Specimen Preparation

#### 2.2.1. Mixture Design

Paste samples were made according to the procedure in [15]. The appropriate mass proportion of Na_2_O to CFA was 10% and dosage of sodium hydroxide is 73 g per 1000 g of fly ash. Modulus ratio of the mixed alkali activator was equal to 1.3 (where modulus ratio = SiO_2_/Na_2_O). The mass ratio of water-to-fly ash was 0.3, 0.32 and 0.35, respectively. The water mass included the water in the sodium silicate and extra water that was used to dissolve the sodium hydroxide. Mortar samples were made with sand to CFA ratio of 2.75. Sodium hydroxide solution and sodium silicate solution had been previously mixed together and heated with water for 5 min so that the Ms would be approximately 1.3. Mixing was done in a laboratory at approximately 23 °C. The geopolymeric fly ash precursor and alkali silicate and hydroxide solution were mixed for 5 min. For mortar, aggregates were then added and followed by a final mixing of another 3 min. Right after the mixing, the fluidity of the fresh geopolymer paste and mortar was determined using the test method for fluidity of cement mortar [19].

#### 2.2.2. Specimen Preparation and Methods for Analysis

After measuring the fluidity, paste was then cast into the cubic molds of the size 20 mm × 20 mm × 20 mm, and mortar was placed in prism molds of the size 160 mm × 40 mm × 40 mm for strength test of paste and mortar, separately, according to [20]. For determination of strength, the samples were then vibrated for 10 s to release any residual air bubbles. Subsequently, samples were sealed with a film to prevent moisture loss and the carbonation of the surface. Samples were demolded after a predetermined delay time of 1day and then put in the oven at the elevated temperature of 70 °C for 24 hours. At the end of this curing period, specimens were put in the laboratory to cool down for 1 hour. The compressive strength results reported were the average of six samples. Microstructural characteristics of the samples were analyzed using X-ray diffraction (XRD) and scanning electron microscope (SEM).

## 3. Results and Discussion

### 3.1. Fluidity

The fluidity of the paste and mortar are shown in Table 1. For the paste, fluidity at the time 30 s and 30 min after mixing were both measured. The fluidity of the geopolymer increased as w/s increased. The geopolymer pastes were more fluid at 30 min than at 30 s. At 30 s after mixing, the alkaline has little effect on the dispersal of fly ash. At 30 min after mixing, the alkaline can contact fully with the surface of fly ash and disperse the fly ash, increasing the fluidity, according to reference [16]. Fly ash particles themselves are spherically shaped, thus allowing easy dispersal within the alkaline environment. Mortars were less fluid than pastes. For the mortar with 0.30 of water-to-fly ash ratio, it is too sticky to obtain the fluidity value. For paste samples, water-to-fly ash ratio from 0.30 to 0.35 may meet the demand of workability as cement materials in construction application. For fly ash-based geopolymer, achieving the target fluidity required less water than needed for Portland cement paste. It was also common sense that more extra water resulted in better fluidity. On the other hand, increases in the concentration of sodium hydroxide decreased the fluidity of the mixes [21]. In this study, all the paste samples consisted of the same amount of fly ash, sodium hydroxide and sodium silicate. Different amounts of extra water were added to dissolve the sodium hydroxide, and the concentration of sodium hydroxide changed. The concentration of sodium hydroxide of pastes with 0.30, 0.32, 0.35 water-to-fly ash ratios was 29.0 M, 22.1 M and 15.3 M, respectively. As a result, the fluidity increased with a decreasing concentration of sodium hydroxide.

**Table 1:** Fluidity of geopolymer with different water-to-fly ash ratio.

Geopolymer	Water-to-fly ash ratio	Fluidity (mm)	
		30 s	30 min
Geopolymer paste	0.30	145	162
	0.32	169	192
	0.35	173	195
Geopolymer mortar	0.30	-	-
	0.32	131	
	0.35	136	

The fluidity of mortar samples was distinctively lower than the paste. In the mortar sample (0.35 of water-to-fly ash ratio), for example, the fluidity value is only 136 mm compared to 173 mm in paste with 0.35 of water-to-fly ash ratio. Sand aggregate hindered the flow of mortar, resulting in a decrease of fluidity. It was noted that for the 40 and 50 wt. % aggregate samples’ workability was low, while geopolymer mortars with 10–30wt. % of aggregate exhibited acceptable fluidity [5]. The synthesis of fly ash-based geopolymer mortar must consider the appropriate mass ratio of sand to fly ash. A previous study revealed that the mass ratio of sand to fly ash could vary from 1.5 to 2.75 [21,22,23]. And 40 wt. % sand samples were considered optimistic [24]. In this study, the mortar had 63 wt. % sand in the sample with 0.35 of water-to-fly ash ratio.

### 3.2. Compressive Strength

**Figure 2:**
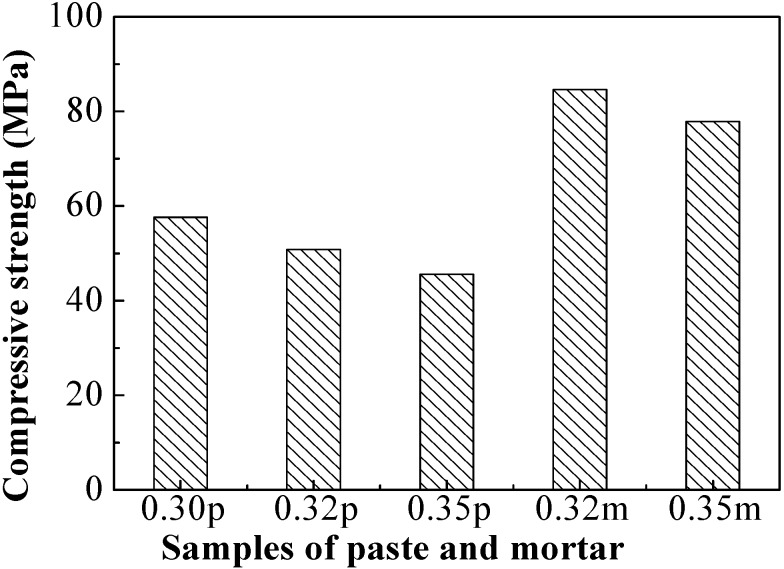
Compressive strength of paste and mortar with different water-to-fly ash ratio.

Compressive strength of paste and mortar with different water-to-fly ash ratios after curing at 70 °C for 24 hours are shown in Figure 2. Average strengths for paste and mortar are 41 MPa and 81 MPa, respectively.

Figure 2 shows that the strength decreased with the increase in water-to-fly ash ratio, a similar behavior as seen in Portland cement. Reduction in compressive strength was observed when additional water was added, thereby indicating the importance of maintaining a high sodium hydroxide concentration. As mentioned previously, the concentration of sodium hydroxide was in excess of 15 M, and a higher concentration sodium hydroxide led to a greater dissolution of the fly ash and consequently increased the degree of geopolymerization and thus also the compressive strength [25]. Moreover, higher solids/liquids ratio contributed to the increase of the porosity level of the hardened geopolymer, causing a decline in strength [26,27]. Normally, alkaline activation of fly ash can be divided into three consecutive stages: (1) decomposition–coagulation; (2) coagulation–condensation; and (3) condensation–crystallization [28]. Water plays an important role in these stages. Water added to dissolve the sodium hydroxide should remain at some level. More extra water leads to a greater amount of the activator. If there was too much activator, the excess would remain in the sample, weakening the structure [29]. Excess added water would stay around the hydrolysis species and hinder the polycondensation, and reactant would reach out from surfaces of geopolymer if cured in water, which may account for the slow strength development [30]. On the other hand, if there was not enough alkaline activator, any aluminosilicate material would have no chance to undergo geopolymerization.

A previous study maintains the opinion that calcium in fly ash would act as a contaminant, forming hydrate assemblages that may decrease mechanical strength and slow down the rate of reaction [6,7,8,9]. High calcium fly ashes show poor reactivity with alkaline activators due to their low glass content and high calcium content, and thus the geopolymer have low strength levels [22]. However, references have recently begun to focus on the use of high calcium fly ash as the precursor of the geopolymer. The high strength of 63 MPa of class C fly ash-based geopolymer paste was obtained by curing at 75 °C for 8 h followed by curing at 23 °C for 28 d [15]. Strength of 65 MPa of coarse high calcium fly ash geopolymer mortar could be obtained with proper delay time and optimum ratio of sodium silicate to NaOH [21]. The highest strength 85MPa in this study also indicated the potential for use of class C fly ash as a geopolymer precursor.

Class C fly ash was produced normally from lignite and sub-bituminous coals and usually contained a significant amount of calcium hydroxide or lime. For making geopolymer, although silica and alumina were the main precursors for the geopolymeric reaction, other factors like curing temperature, water content, concentration of the alkaline compound also played a significant role in the resultant compressive strength. And a larger amount of silica and alumina did not directly relate to a higher strength. Thus, despite class C fly ash’s lower content of silica and alumina, high strength could also be obtained when other factors affecting compressive strength remained in an optimistic level.

For paste, compressive strength depends on the strength of the geopolymer gel. It was obvious that at elevated curing temperature, the aluminosilicate geopolymer was the main contributor to hardening behavior. For mortar, however, as aggregates were added, compressive strength was then affected by many factors, not only the geopolymer binder, but also the interfacial bonding between the geopolymeric gel and aggregate and aggregate itself [5]. Addition of up to 63 wt. % sand aggregate may lead to a more compacted structure compared to the paste. Two reasons that may contribute to mortar’s higher strength may be (1) potential partial reaction of the surfaces of siliceous aggregates with the alkali silicate solution forming additional reaction products surrounding the aggregate particles; and (2) a highly dense and uniform binder–aggregate interface. A detailed study in the XRD patterns and SEM analysis thus remained.

### 3.3. Microstructure

#### 3.3.1. XRD Analysis

**Figure 3:**
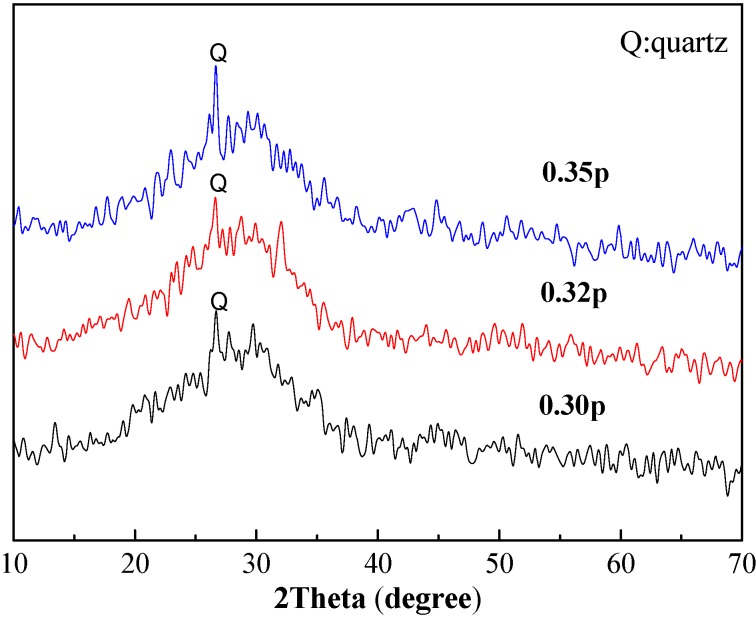
X-ray diffraction of the paste with 0.30, 0.32 and 0.35 of water-to-fly ash ratio.

**Figure 4:**
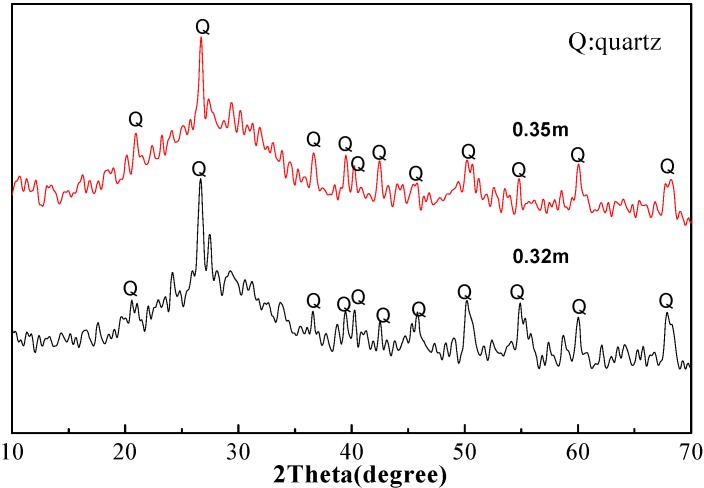
X-ray diffraction of the mortar with 0.32 and 0.35 of water-to-fly ash ratio.

XRD patterns for paste and mortar with different water-to-fly ash ratio are shown in Figure 3 and Figure 4. Results revealed that a large part of the structure was amorphous. All samples showed a broad band rather than sharp diffraction peaks around 30 degrees of 2theta, showing that gels have been formed in these samples. Additionally, peaks of quartz from inactivated raw material had been determined.

From previous studies of in alkali-activated fly ashes, different hydrate assemblages were formed. Besides gel-like sodium aluminosilicate hydrates (N-A-S-H), zeolites were the main hydration products depending on mix composition and curing regime [31,32,33,34,35]. N-A-S-H gels were difficult to characterize with XRD due to their amorphous or nano-crystalline nature [36]. For class C fly ash-based geopolymer, the governing reactions became more complex in the presence of soluble Ca species. Compared to the low calcium fly ash, the activated high calcium fly ashes exhibited a different composition of the hydrate assemblages [22], and the role of calcium during this process was of significant practical interest. One important factor when working with high calcium fly ash was the location of the glass diffraction maximum, the highest point in the broad band in the X-ray pattern [14]. Calcium species of oxides and silicate minerals from class C fly ash could either (1) precipitate as Ca(OH)_2_; (2) be bonded in geopolymeric gel by replacing cations within the geopolymer; or (3) react with dissolved silicate and aluminate species to form C–S–H gel [28]. Based on published results, noticeable amounts of crystalline or a poorly crystalline C–S–H phase was usually formed at higher Ca concentrations [37]

#### 3.3.2. SEM Analysis

**Figure 5:**
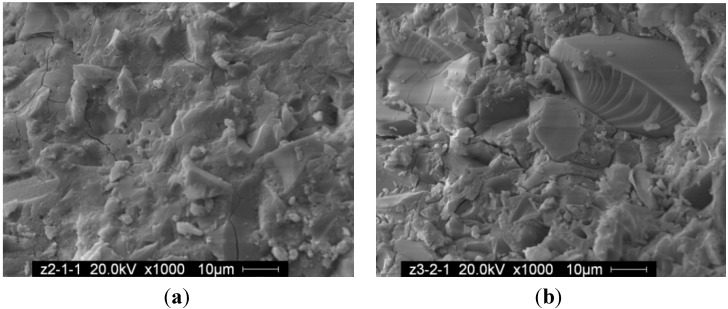
SEM of paste with W/F (**a**) 0.32; (**b**) 0.35.

**Figure 6:**
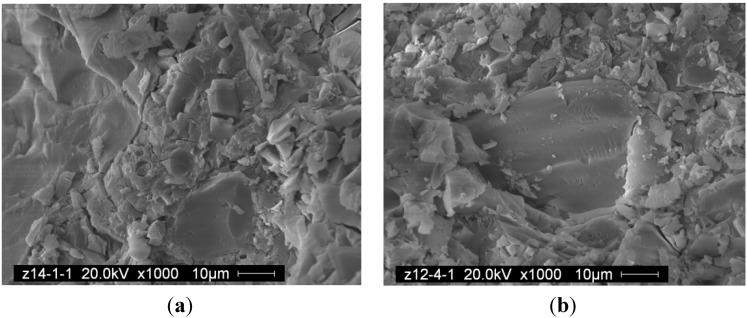
SEM of mortar with W/F (**a**) 0.32; (**b**) 0.35.

SEM micrographs of paste and mortar with different water-to-fly ash ratio are shown in Figure 5 and Figure 6. SEM revealed that the paste and mortar have formed a relatively dense reacted product regardless of the water-to-fly ash of paste or mortar. Cracks present in the geopolymer gel were believed to occur during testing for compressive strength or when the sample was placed in vacuum for coating for SEM. The compacted structure of mortar in this study indicated that there was a great deal of reacted product corresponding to high compressive strength.

## 4. Conclusions

In this paper, mechanical properties and microstructural characteristics of the class C fly ash-based geopolymer paste and mortar were studied. The experimental and analytical studies led to the following conclusions:

(1) For class C fly ash-based geopolymer paste, low W/F could meet the demand for an approving fluidity. Increases in the concentration of sodium hydroxide decreased the fluidity of the mixes. As sand aggregates were added, fluidity decreased distinctly;
(2) Class C fly ash-based geopolymer paste and mortar could both obtain high compressive strength after curing at 70 °C for 24 h. Compressive strength of mortar was much higher than paste with the same water-to-fly ash ratio;
(3) In the XRD patterns of the paste and mortar samples, broad band diffraction peaks around 30 degrees of 2theta show that gels have been formed in these samples. SEM analysis revealed that the dense structures of mortar and paste contribute to the high compressive strength.
